# Coping Strategies and Considering the Possibility of Death in Those Bereaved by Sudden and Violent Deaths: Grief Severity, Depression, and Posttraumatic Growth

**DOI:** 10.3389/fpsyt.2020.00749

**Published:** 2020-08-06

**Authors:** Joscelyn E. Fisher, Jing Zhou, Rafael F. Zuleta, Carol S. Fullerton, Robert J. Ursano, Stephen J. Cozza

**Affiliations:** ^1^ Henry M. Jackson Foundation for the Advancement of Military Medicine, Bethesda, MD, United States; ^2^ Center for the Study of Traumatic Stress, Department of Psychiatry, Uniformed Services University of the Health Sciences, Bethesda, MD, United States

**Keywords:** bereavement, coping, depression, grief, posttraumatic growth, preparedness, possibility of death

## Abstract

**Background:**

Bereavement by sudden and violent deaths can lead to increased grief severity, depression, and reduced posttraumatic growth compared to those bereaved by natural causes. These outcomes can be affected by coping strategies and whether a survivor had been “prepared” for the death. The present study examined the effect of coping and considering the possibility of death on grief severity, depression, and posttraumatic growth in those bereaved by sudden deaths.

**Methods:**

Participants bereaved by suicide, accident, or combat deaths completed an online survey about demographics (including the cause of death), coping, grief severity, depression, and posttraumatic growth. A factor analysis of the coping measure yielded factors representing three coping strategies: avoidant coping, supportive coping, and active coping. These three strategies, the causes of death and considering the possibility of death were used as predictors of either grief severity, depression, or posttraumatic growth in multivariate linear regression models.

**Results:**

Each coping strategy and cause of death was differentially associated with grief severity, depression, and posttraumatic growth. Specifically, supportive coping and active coping were each only associated with higher posttraumatic growth. In contrast, avoidant coping was associated with all outcomes (higher grief severity and depression and lower posttraumatic growth). In addition, accidents and suicides (compared to combat deaths) had independent effects on grief severity and posttraumatic growth. Considering the possibility of death interacted with avoidant coping and also with supportive coping to predict grief severity in combat-loss survivors.

**Discussion:**

Findings highlight the differential contributions of coping strategies and their complex relationships with cause of death in contributing to grief severity, depression, and posttraumatic growth. Avoidant coping contributed to negative outcomes and inhibited posttraumatic growth, suggesting its importance as a target for therapeutic intervention. Although supportive and active coping facilitated posttraumatic growth, they had less of a role in mitigating grief severity or depression in this study. Although considering the possibility of death appeared to mitigate negative outcomes among survivors of combat death, avoidance of that possibility is likely protective for the majority of family members whose loved ones return home safely.

## Introduction

Bereavement is a common and universal stressor that sometimes leads to complicated grief or depression (e.g., 1). These outcomes occur more frequently for those who have been bereaved by sudden and violent deaths, such as accidents, suicides, or military combat deaths (2). Although some studies identified differences in psychological health between survivors of suicide loss and other causes of death (3–5), other studies reported minimal or no differences (6–9).

These potential differences in mental health outcomes could be attributed to coping strategies used following a death. Coping has been defined and classified in various ways (10–12). Many studies have operationalized coping after bereavement by using either the COPE (10) or Brief COPE (13), a self-report measure that assesses 14 coping strategies (14–24). These coping strategies (as measured by COPE or Brief COPE subscales) were grouped by Schnider et al. (24) into three categories: *problem-focused* coping (active coping, planning, instrumental support, and religion), *active* emotional coping (venting, positive reframing, humor, acceptance, and emotional support), and *avoidant* emotional coping (self-distraction, denial, behavioral disengagement, self-blame, and substance use). This three-group structure is consistent with several factor analyses of the COPE and Brief COPE in non-bereaved samples (25–29). However, the combinations of individual subscales that defined the three factors has differed between studies, leading to variations in coping strategy labels [e.g., Engagement, Disengagement, and Help-Seeking in (25); Active Coping, Repressive Coping, and Affective Coping in (22)]. Alternate coping inventories employed in other studies of bereaved samples have similarly yielded three-dimensional coping models [e.g., task-oriented, emotion-oriented, and avoidance-oriented coping; (30)].

Despite the variety of instruments and labels employed, associations between certain types of coping strategies and distinct outcomes have been consistent in bereaved samples. Problem-focused coping (16) has been associated with posttraumatic growth in suicide loss survivors, and affective coping has been associated with lower post-traumatic stress disorder (PTSD) symptom scores (21). Avoidant (or repressive) coping has been associated with increased severity of negative outcomes, such as grief (17–19, 24), PTSD (22), mental distress (20), and depression (18), but has also been associated with lower grief severity (30), suggesting that the relationship between avoidant coping and grief severity is complex and likely affected by additional factors related to the death.

As many studies of bereavement coping strategies either combined causes of death or looked at one cause of death alone (e.g., suicide), there is limited information about the effects of different coping strategies according to specific types of sudden and violent deaths. Use of avoidant coping (31) and decreased use of support from others (32, 33) have been reported in survivors of suicide loss, but to our knowledge, no studies have reported about coping strategies specifically in accidental deaths. Although there have been no published research studies about coping strategies used following combat deaths, anecdotal reports suggest that combat-loss survivors are more likely to receive community support, feel a sense of pride in their family member’s sacrifice, and find meaning after the death compared to other sudden and violent deaths. These reports might translate to greater use of supportive and active (e.g., positive reframing) coping strategies compared to suicide-loss or accident-loss survivors.

In addition to the effect of coping strategies, the unexpected nature of sudden and violent deaths is also likely to contribute to increased risk of mental health difficulties and reduced well-being. For example, unexpected deaths and perceptions of less preparedness for the death have both been associated with complicated grief (34), increased depression (35, 36), and other mental health disorders (36). Other variables related to the nature of a death (i.e., violent, extent of suffering, prolonged dying process, opportunity to say goodbye) have been associated with “bereavement intensity”, but being unprepared for a death was the strongest predictor of this association (14).

Despite the usefulness of the construct and the importance of its associations, the term “preparedness” is ambiguous. For example, even when deaths are expected, survivors may not feel “prepared” for a death. Additionally, preparedness may refer to logistical, as well as psychological readiness for the death. A more precise indication of psychological readiness may be whether the individual considered the possibility of death, as it more clearly refers to a psychological process. Different causes of sudden deaths (combat deaths vs. accidents vs. suicide) would be expected to vary in the degree to which the possibility of death was considered. For instance, death is a known risk associated with military combat operations, so surviving family members whose service member died (either due to combat, accident or suicide) while deployed on a combat mission would likely have considered such a possibility. Survivors of some suicide deaths may have considered the possibility of death, especially if attempts had been previously made or if the death followed a period of suffering from a mental illness. In contrast, survivors of accidents are the least likely to have considered such a possibility since accidents, by definition, are likely to be unpredictable. Whether a bereaved individual considered the possibility of death prior to its occurrence combined with the coping style used after the death could explain differences in mental health outcomes between those bereaved by deaths due to combat, suicide, and accidents.

The current study examined the effects of coping strategies and considering the possibility of death on grief severity, depression, and posttraumatic growth in survivors bereaved by suicide, accident, and combat. It was hypothesized that suicide-loss survivors would be more likely to use avoidant coping and less likely to use support-type strategies, resulting in higher grief severity and depression, and reduced positive outcomes compared to accident-loss and combat-loss survivors. In comparison, combat-loss survivors would use more supportive coping and/or active coping strategies, which would be associated with more posttraumatic growth and lower grief severity. Survivors of combat death were also expected to have been more likely to have considered the possibility of death compared to survivors of suicides and accidents, given that all combat deaths occurred during combat deployment compared to smaller percentages of accidents and suicides that occurred during combat deployment. This combination of coping and considering the possibility of death would contribute to less adverse and more positive outcomes.

## Methods

### Participants

Surviving parents, spouses/partners, siblings, and adult children of U.S. military service members who died by combat, accident, or suicide between September 11, 2001 and January, 2014 were enrolled as part of the National Military Family Bereavement Study (NMFBS^1^), a study of the impact of military service member death on their family members. Participants were recruited for the NMFBS through grief support organizations, online advertisements, and word-of-mouth and provided informed consent after receiving a description of the study. Analyses for this manuscript included: 328 survivors bereaved by suicide, 384 survivors bereaved by accidents, and 997 survivors bereaved by combat deaths (total *n* = 1,709). Time since death ranged from under 6 months to over 12 years (mean = 4.89 years; mode = 3 years), though most participants (65%) had been bereaved between 1 and 7 years at the time of assessment. One hundred thirty-one family members were bereaved less than 1 year (34 of these 131 were bereaved less than 6 months).

### Design

Participants provided online consent and completed an online survey located on the NMFBS website^1^. The survey assessed demographics, information about relationship to the service member, circumstances of the death, and physical and psychological reactions and was designed for participants to complete in 30–45 min. Eighty-three percent of participants who consented to participate then completed the study. The study was conducted in accordance with ethical standards as approved by the Human Research Protection Program in the Office of Research at the Uniformed Services University of the Health Sciences and all analyses were conducted on de-identified data.

### Measures

The following measures were analyzed for the present study.

*Brief COPE* (13). (28 items): 14 subscales: 1) Active Coping, 2) Planning, 3) Positive Reframing, 4) Acceptance, 5) Humor, 6) Religion, 7) Using Emotional Support, 8) Using Instrumental Support, 9) Self-Distraction, 10) Denial, 11) Venting, 12) Substance Use, 13) Behavioral Disengagement, and 14) Self-Blame. The overall scale has good performance characteristics. Instructions asked how the participant has been coping with “stress in your life since the death of your service member”.

*Inventory of Complicated Grief* (ICG; 37) is a 19-item self-report measure of clinically impairing grief symptom severity during the last month. The ICG has been widely used as a screening tool to assess grief severity (38–40).

*Patient Health Questionnaire* (PHQ-9; 41) is a 9-item measure that has been used as a reliable measure of depression in medical and general population settings (41). Participants were asked how often they had been bothered by symptoms during the previous 2 weeks.

*Posttraumatic Growth Inventory*—*Short Form* (PTGI-SF; 42) is a measure of positive changes or growth after traumatic events assessed on a six-point Likert scale. A 10-item short form of the PTGI was used in this study. Participants responded to each item according to whether it was true as a result of the death of their family member.

*Possibility of death*: One item assessed the frequency with which participants considered the possibility of their loved one’s death. Participants chose one of the following five options to complete the stem: “*During the month prior to my service member’s death…*”: (1) I *never* thought about the possibility of my service member dying; (2) I thought about the possibility of my service member dying *1–3 times during the month (or about once or twice every 2 weeks)*; (3) *once each week*; (4) *3–5 times each week*; (5) *every day.* Responses to this item were dichotomized as “never thought about the possibility” (i.e., 1) and “thought about the possibility” (i.e., 2–5) for the present study analyses.

### Data Analysis

Descriptive statistics (means, standard deviations, frequency distributions, crosstabs, etc.) of the study participants’ demographics and other characteristics were examined. In order to determine how coping strategies clustered among bereaved family members within the sample, an exploratory factor analysis (EFA) was conducted. The EFA of the 14 Brief COPE subscales supported a three-factor structure (eigenvalue greater than 1). These factors were labeled *supportive*, *avoidant*, and *active* coping. A confirmatory factor analysis (CFA) was then conducted based on the EFA and prior literature to determine whether the coping subscales factored into three factors representing supportive, avoidant, and active coping strategies. The supportive coping factor consisted of two coping subscales: emotional support (factor loading: 0.80) and instrumental support (factor loading: 0.85). The avoidant coping factor consisted of three coping subscales: denial (factor loading: 0.60), behavioral disengagement (factor loading: 0.72), and self-blame (factor loading: 0.61), and the active coping factor consisted of three coping subscales: active coping (factor loading: 0.80), positive reframing (factor loading: 0.54), and planning (factor loading: 0.73). Indices of model fit indicated that the CFA model had a good fit [comparative fit index (CFI) = 0.92, standardized root mean square residual (SRMR) = 0.06, root mean square error of approximation (RMSEA) = 0.10]. Coping subscales in each resulting factor were identified and then summed to create a continuous score that represented each coping strategy. Each factor score was standardized by dividing the number of subscales in the factor. As these three factors are conceptually similar to other studies that derived a three-factor structure from the COPE and Brief COPE (25–29), these standardized factor scores were included in subsequent regression analyses as indices of coping strategies.

To examine the effects and interactions of coping strategies, cause of death (suicide, accidents, and combat), and possibility of death (never thought about possibility of death vs. thought about the possibility) on grief severity, depression, and posttraumatic growth, multivariate linear regression analyses were performed. Linear splines were applied to examine the relationship between time since death and outcomes when appropriate. In order to adjust for the cluster effects of participants nested within families, generalized estimating equations were used to fit linear regressions in all analyses (43). Model assumptions were checked using histograms, normal probability plots, scatter plots, smooth spline, plots, and other model diagnostics. Potential confounding variables (i.e., time since death, relationship type, participant age, gender) were also included in the models.

All statistical analyses were performed using SAS 9.4 (SAS Institute Inc., Cary, North Carolina) and statistical significance was defined as *p* < 0.05 using two-tailed tests.

## Results

### Participant Characteristics

Demographic characteristics of the study participants, causes of service member death, relationship to the deceased, coping strategy factor summary scores, thoughts about the possibility of death, and means and standard deviations of the ICG, PHQ-9, and PTGI-SF scores are presented in **Table 1**.

**Table 1:** Demographics and other characteristics of study participants.

Characteristics	Total (*N* = 1709)	
	*N* or M	% or SD
Age in years (M and SD)	46.98	13.26
		
Gender		
Male	358	20.97%
Female	1349	79.03%
		
Cause of death of DSM*		
Combat related	997	58.34%
Accident	384	22.47%
Suicide	328	19.19%
		
Time since death in years (M and SD)	4.89	2.87
		
Relationship to the deceased		
Parent	965	56.57%
Spouse	351	20.57%
Sibling	345	20.22%
Adult child	45	2.64%
		
Possibility of death		
Never thought about possibility of death	740	44.15%
Thought about possibility of death	936	55.85%
		
Coping factor summary scores (M and SD, range = 2–8)		
Supportive	4.84	1.69
Avoidant	3.44	1.32
Active	4.97	1.54
		
ICG total score (M and SD)	25.42	14.98
		
PHQ total score (M and SD)	8.31	6.79
		
PTGI total score (M and SD)	25.15	12.80

*Deceased service member.

DSM, Deceased service member; ICG, Inventory of Complicated Grief; PHQ, Patient Health Questionnaire; PTGI, Posttraumatic Growth Inventory.

### Possibility of Death and Cause of Death

More than half (56%) of the participants thought about the possibility of their service member’s death. The percentages differed between each of the causes of death (*p* < 0.05). The percentages were much higher among combat-loss survivors (70%) compared to accident-loss survivors (41%) and suicide-loss survivors (29%).

To determine whether being deployed at the time of death was associated with survivors considering the possibility of death, the above was stratified according to deployment status at the time of death. Twenty-three percent of accidental deaths and 8% of suicide deaths occurred during combat deployment. Of survivors of accidents and suicide whose loved one was combat-deployed at the time of death, 64% and 42%, respectively considered the possibility of death, compared to 34% and 28% of respective survivors of accidents and suicides whose loved one was not combat-deployed at the time of death. In contrast, 70% of combat survivors thought about the possibility of death.

### Possibility of Death and Outcomes

Participants who never thought about the possibility of their service member’s death had significantly higher grief severity total scores (mean = 26.1, standard deviation = 15.8) than those who had thought about the service member’s death (mean = 24.6, standard deviation = 14.2, *p* = 0.04). There were no significant differences in either depression or posttraumatic growth between participants who never thought about their service member’s death and those who did.

### Coping Strategies and Cause of Death

Means of each coping strategy within each cause of death were compared. Suicide-loss survivors had significantly higher mean avoidant coping scores (mean = 3.7, standard deviation = 1.3) compared to combat-loss survivors (mean = 3.3, standard deviation = 1.3, *p* < 0.05), but not accident-loss survivors (mean = 3.5, standard deviation = 1.3). Supportive coping and active coping strategies did not differ according to cause of death.

### Coping Strategies and Cause of Death Predicting Outcomes

To examine the effects of coping strategies and causes of death on grief severity, depression, and posttraumatic growth (*N* = 1709), three separate multivariate linear regression models were conducted in which all coping strategies and all causes of death were entered as predictors (**Table 2**). Avoidant coping predicted grief severity. The second model indicated that avoidant coping was also associated with depression. The third model indicated that all coping strategies predicted posttraumatic growth; avoidant coping was negatively associated with posttraumatic growth, while active coping and supportive coping were each positively associated with posttraumatic growth. In addition, accident deaths and suicide deaths were associated with lower posttraumatic scores compared to combat deaths. There were no significant interactions between any of the coping strategies and causes of death in any of the three models.

**Table 2:** Coping strategies and cause of death predicting grief, depression and posttraumatic growth (*N* = 1709)*.

Predictors	ICG Total Score		PHQ Total Score		PTGI Total Score	
	Estimate (SE)	*p*-value	Estimate (SE)	*p*-value	Estimate (SE)	*p*-value
Coping strategies						
Supportive coping	0.03 (0.19)	0.87	−0.14 (0.09)	0.13	**0.89 (0.20)**	**<0.01**
Avoidant coping	**7.29 (0.22)**	**<0.01**	**3.03 (0.11)**	**<0.01**	−**1.41 (0.23)**	**<0.01**
Active coping	0.27 (0.22)	0.21	0.05	0.61	**2.99 (0.21)**	**<0.01**
Cause of death						
Combat related	Ref		Ref		Ref	
Accident	0.95 (0.68)	0.16	−0.25 (0.33)	0.43	−**1.85 (0.72)**	**0.01**
Suicide	−**1.76 (0.73)**	**0.02**	0.26 (0.41)	0.52	−**1.94 (0.79)**	**<0.01**

*Models adjusted for participants age, gender, relationship with service member (parent, spouse, sibling or adult child), and time since death.

ICG, Inventory of Complicated Grief; PHQ, Patient Health Questionnaire; PTGI, Posttraumatic Growth Inventory. The text in bold indicates a p-value less than .05.

### Possibility of Death, Coping Strategies, and Interactions Predicting Outcomes

To examine the effect of considering the possibility of death on grief severity, depression, and posttraumatic growth, three additional regression models were conducted. These models added the possibility of death as an additional predictor to the models that contained cause of death and the three coping strategies. As described above, all coping strategies predicted posttraumatic growth and avoidant coping predicted higher scores on grief severity and depression measures. Considering the possibility of death was not associated with any of the three outcomes (grief: *p* = 0.95; depression: *p* = 0.053; posttraumatic growth *p* = 0.71).

**Figure 1:**
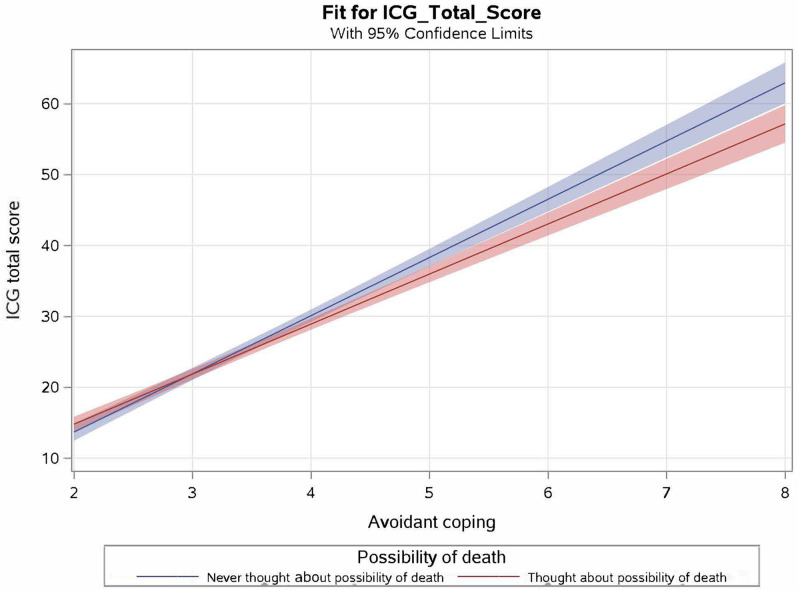
Interaction of avoidant coping and possibility of death predicting grief severity in the total sample. Plot reflects regression model containing avoidant coping, possibility of death and their interaction predicting ICG total score.

Interactions between the possibility of death and each coping strategy were then tested. None of the interactions between possibility of death and any of the three coping strategies predicted posttraumatic growth or depression. However, the interaction between possibility and avoidant coping predicted grief severity (**Table 3** and **Figure 1**). The interaction suggests that in general, more avoidant coping was associated with increases in grief severity. However, for those who never considered the possibility of death, increased use of avoidant coping was associated with higher increases in grief severity than those who did consider the possibility.

**Table 3:** Interactions between possibility of death and coping strategies predicting grief (*N* = 1,709)*.

Predictors	ICG Total Score	
	Estimate (SE)	*p*-value
Possibility of death * supportive coping		
Never thought about possibility of death	0.45 (0.37)	0.23
Thought about possibility of death	Ref	Ref
Possibility of death * avoidant coping		
Never thought about possibility of death	**1.08 (0.42)**	**0.01**
Thought about possibility of death	Ref	Ref
Possibility of death * active coping		
Never thought about possibility of death	0.56 (0.42)	0.18
Thought about possibility of death	Ref	Ref
Cause of death		
Combat related	Ref	
Accident	0.99 (0.69)	0.15
Suicide	−**1.74 (0.77)**	**0.02**

*Model adjusted for participants age, gender, relationship with service member (parent, spouse, sibling or adult child), and time since death.

ICG, Inventory of Complicated Grief. The text in bold indicates a p-value less than .05.

### Possibility of Death, Coping, and Interactions Stratified by Cause of Death

**Figure 2:**
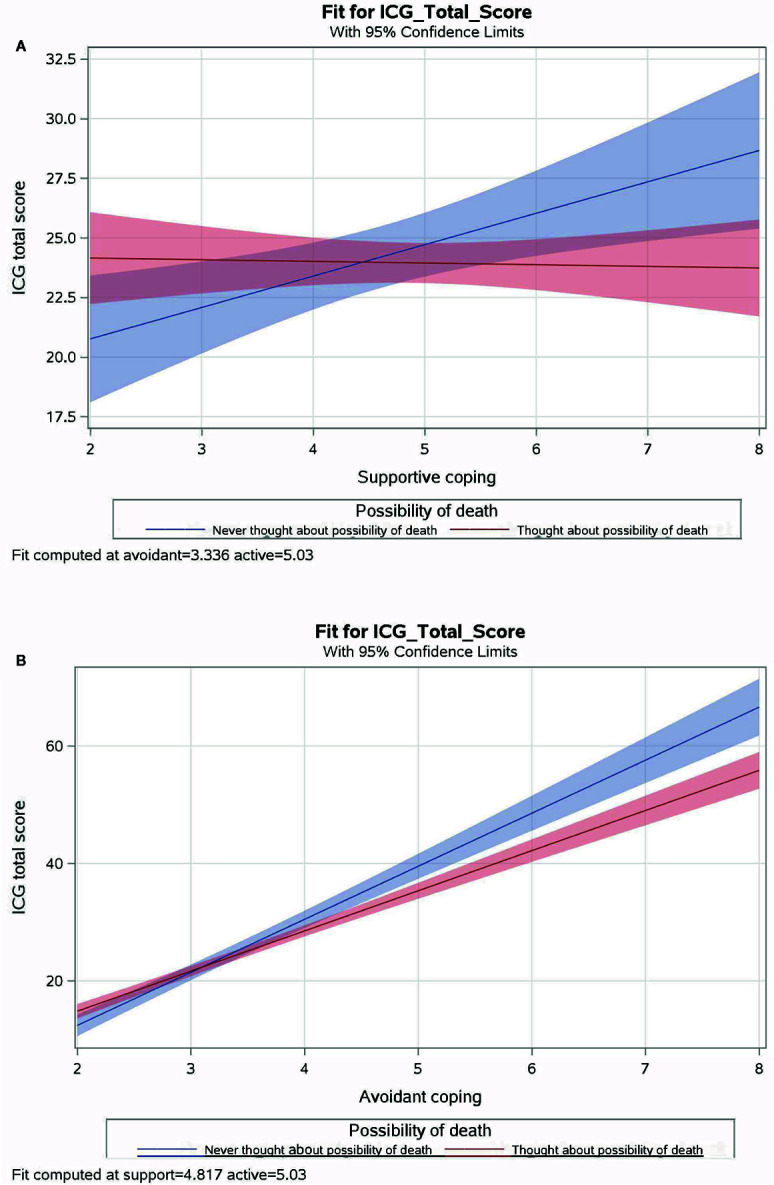
Interactions between coping strategies and possibility of death predicting grief severity in combat-bereaved participants. Note: Plots reflect a regression model of three coping strategies (supportive coping, avoidant coping, active coping strategies), considering the possibility of death and three interactions (coping X possibility) predicting total ICG score in combat-bereaved participants. The interaction between active coping and possibility of death was not plotted because it was not significant at the 0.05 level.

In order to further examine the interactive effect of possibility of death and avoidant coping on grief severity according to different causes of death, the regression analyses described above were repeated with the addition of stratifying by cause of death. There were no significant interactions between the possibility of death and coping strategies predicting grief severity in accident bereaved or suicide bereaved. However, the interaction between considering the possibility of death and supportive coping predicted grief severity in combat-bereaved individuals (*N* = 997). For those who never thought about the possibility of death, more supportive coping was associated with increased grief severity scores. However, for those who did consider the possibility of death, grief scores did not vary according to the amount of supportive coping (**Table 4** and **Figure 2A**).

**Table 4:** Interactions between possibility of death and coping strategies predicting grief in combat-bereaved (*N* = 997)*.

Predictors	ICG Total Score	
	Estimate (SE)	*p*-value
Possibility of death * supportive coping		
Never thought about possibility of death	**1.47 (0.53)**	**0.01**
Thought about possibility of death	Ref	Ref
Possibility of death * avoidant coping		
Never thought about possibility of death	**2.24 (0.59)**	**<0.01**
Thought about possibility of death	Ref	Ref
Possibility of death * active coping		
Never thought about possibility of death	0.25 (0.59)	0.67
Thought about possibility of death	Ref	Ref

*Model adjusted for participants age, gender, relationship with service member (parent, spouse, sibling or adult child), and time since death.

ICG, Inventory of Complicated Grief. The text in bold indicates a p-value less than .05.

The interaction between considering the possibility of death and avoidant coping also predicted grief severity for combat-bereaved participants. For those who never considered the possibility of death, more avoidant coping was associated with increases in grief severity than those who did consider the possibility (**Table 4** and **Figure 2B**).

## Discussion

The present study investigated the relationships between causes of death, coping strategies, whether participants considered the possibility of death, and post-bereavement outcomes, including grief severity and depression, as well as posttraumatic growth. In summary, the findings indicated that each type of coping strategy was differentially associated with these three outcomes. Specifically, avoidant coping had broad and negative effects on post-bereavement outcomes by worsening grief severity and depression, and decreasing posttraumatic growth. In contrast, neither supportive coping nor active coping were associated with either grief severity or depression, but were associated with higher levels of posttraumatic growth, suggesting unique pathways for supporting growth within the population of those who have been suddenly and violently bereaved. In addition to these effects of coping strategies on outcomes, accidents and suicides (compared to combat deaths) had independent effects on grief severity and posttraumatic growth. These findings imply that there are circumstances unique to causes of death, which are independent of coping strategies, that contribute to these outcomes. It was anticipated that whether a survivor had considered the possibility of death might account for differences between combat, suicide, and accident-related deaths. Although a higher percentage of combat-loss survivors had considered the possibility of death compared to accident-loss survivors and suicide-loss survivors, this variable alone did not account for additional variance in outcomes when coping strategies were included. However, the interaction between considering the possibility of death and specific coping strategies did account for grief severity among combat-death survivors. Implications of these findings are discussed below.

Each type of coping strategy and cause of death was differentially associated with grief severity, depression, and posttraumatic growth. Specifically, supportive coping and active coping were each associated with higher posttraumatic growth, and avoidant coping was associated with lower posttraumatic growth. In contrast, supportive coping and active coping were not associated with either depression or grief severity, though avoidance was associated with grief severity and depression. These results are consistent with other studies that found multiple coping strategies associated with posttraumatic growth in bereaved individuals (44), especially active coping strategies (45). However, findings also raise interesting questions about therapeutic coping-focused approaches that intend to minimize grief severity and depression, and suggest that emphasis on avoidant coping, rather than focusing on supportive or active coping, would better address those outcomes.

The substantial negative effect of avoidant coping on all three post-bereavement outcomes (higher grief and depression and lower posttraumatic growth) in this study was striking and is consistent with prior studies (17–19, 24). Though avoidant coping was strongly and negatively associated with grief severity, suicide deaths also accounted for additional variance in grief severity. A direct comparison of grief severity scores by cause of death (without age, gender, time since death, relationship covariates) indicated that suicide and accident survivors had *higher* grief severity compared to combat survivors. However, when coping strategies (and covariates) were taken into account, avoidant coping, in particular, accounted for the variance associated with higher grief severity. In fact, the variance associated with suicide-loss was unexpectedly associated with *lower* grief severity in this model. These findings suggest that negative effects of suicide on grief severity described in the literature may be accounted for by the effect of avoidance rather than any unique characteristic of suicide deaths. This result is consistent with a previous study that also found that avoidant coping fully mediated the effect between suicide bereavement and grief (31). This relationship between avoidance and grief is consistent with the clinical literature that has characterized avoidance as a symptom of a disorder of prolonged and impairing grief (46) and a target of evidence-based interventions for this grief disorder (47, 48).

In addition to the effects of coping strategies, cause of death was distinctly related to outcomes, in that accident and suicide deaths (compared to combat deaths) had independent effects on grief severity and posttraumatic growth. This pattern of results implies that there are circumstances unique to causes of death that are independent of coping strategies which impact these outcomes and warrant further investigation. It was anticipated that one of the characteristics that differs between causes of death might be whether a survivor had considered the possibility of death. Indeed, considering the possibility of a family member’s death was differentially associated with each cause of death and with each outcome when examined alone, (i.e., without accounting for coping strategies). As hypothesized, 70% of combat-loss survivors considered the possibility of death, likely due to the known risk of combat operations. In contrast, only 29% of suicide-loss survivors considered the possibility of death. This pattern may be related to stigma associated with self-disclosure among those having thoughts of suicidal ideation (49, 50), or that even when informed that someone is struggling with emotional distress or mental illness, family members may deny the possibility of suicide as a potential outcome. Never considering the possibility of death was associated with higher grief severity than those who did consider it, consistent with Barry et al. (34) who found the same relationship between grief and preparedness.

However, considering the possibility of death did not account for additional variance in outcomes when coping strategies were included. Instead, the interaction between considering the possibility of death and specific coping strategies accounted for grief severity. Specifically, considering the possibility of death interacted with avoidant coping to predict grief severity. Though this interaction was significant in the full sample, when the sample was stratified by cause of death, it was only significant for combat-loss survivors. The interaction indicated that, overall, more avoidant coping was associated with increased grief severity. However, for those who never considered the possibility of death, increased use of avoidant coping was associated with even higher increases in grief severity compared to those who did consider the possibility. In other words, those who reported not considering the possibility of death and also engaged in avoidant coping (defined in this study as denial, behavioral disengagement, and self-blame) experienced an additive effect which led to higher grief severity. In fact, not considering the possibility of death may itself be an avoidant strategy. For family members of military service members who are involved in combat, such an avoidant strategy may be a healthy way to deny the risks that their loved one is engaging in. However, in the event of death, avoidance of the thought of death while employing continued avoidance coping would explain its additive effect and is consistent with evidence that avoidance behaviors prevent grief integration and lead to prolonged and impairing grief (51).

Considering the possibility of death also interacted with supportive coping to predict grief severity in combat-loss survivors. For those who never thought about the possibility of death, more supportive coping was associated with higher grief severity. At first glance this finding may appear confusing—why would supportive coping result in higher grief severity? However, these findings are associative rather than causal. An explanation of these results is that combat-bereaved survivors who never considered the possibility of the death of their loved ones were more likely to have higher grief severity, under which circumstances they more actively engaged supportive coping. That we identified this relationship in combat-death survivors but not survivors of suicide or accidents may be due to the larger number of combat-death survivors in our sample. However, it could also be explained by the greater availability of community and grief support among combat-death survivors, especially compared to suicide, which is often associated with stigmatization and isolation (3).

Findings of the study should be interpreted in context of its limitations and strengths. Its cross-sectional design does not allow conclusions regarding causal mechanisms between coping strategy and grief severity, depression, or posttraumatic growth. In addition, the item used to assess consideration of the possibility of death required retrospective recall and may be subject to bias or inaccuracies, especially given the wide range of time since loss within the sample. It may be more precise to interpret this variable as the participant’s perception of whether the possibility of death was considered. In addition, the sample consisted of approximately 80% female participants, so findings may not be as generalizable to males. Despite these limitations and to our knowledge, this is the first study to investigate the relationship between considering the possibility of death (or preparedness) and coping strategies. It is also the first to report on coping strategies used by combat-loss survivors compared to suicide-loss and accident-loss survivors. In addition, the large sample size allowed examination of distinct effects of each of the three coping strategies in combination with considering the possibility of death on grief severity, depression, and posttraumatic growth.

In conclusion, study findings highlight the differential contributions of coping strategies and their complex relationships with cause of death in contributing to grief severity, depression, and posttraumatic growth. Consistent with the existing literature, avoidant coping, in particular, was a potent contributor to negative outcomes, as well as an inhibitor of posttraumatic growth, suggesting its importance as a target for therapeutic intervention. Although supportive and active coping facilitated posttraumatic growth, they had less of a role in mitigating grief severity or depression in this study. These findings are consistent with therapeutic targets described in evidence-based programs (48, 52), and suggest that targeting coping strategies in community support programs or therapeutic interventions can minimize grief severity and promote posttraumatic growth among bereaved military family members. Specifically, efforts to decrease avoidance (e.g., accepting the reality of the death, enhancing behavioral and social engagement) and to increase supportive and active coping (e.g., seeking and accepting assistance, developing meaningful understanding of the death) should be recommended approaches to enhance survivors’ grief adaptation. Considering the possibility of death appeared to mitigate negative outcomes among survivors of combat death. Although this finding might suggest the importance of preparing military family members for the possibility of death, avoidance of that possibility is likely protective for the vast majority of family members whose loved ones return home safely.

## Data Availability Statement

The datasets presented in this article are not readily available because they are restricted due to privacy reasons. Requests to access the datasets should be directed to the Principal Investigator, National Military Family Bereavement Study (SC, stephen.cozza@usuhs.edu). Requests to access this data will be decided at the discretion of the Principal Investigator and the Uniformed Services University based upon the scientific merits of any proposed project.

## Ethics Statement

The studies involving human participants were reviewed and approved by institutional review board at the Uniformed Services University of the Health Sciences. The participants provided their written informed consent to participate in this study.

## Author Contributions

JF conceptualized the presented ideas and developed them in consultation with JZ and SC. JZ performed the data analyses and SC provided guidance. JF wrote the manuscript with substantial contributions from JZ and SC. RZ assisted with the preparation of the final version of the manuscript. CF and RU provided leadership.

## Funding

This paper represents research funded by the Department of Defense—Congressionally Directed Medical Research Programs (W81XWH-11-2-0119) and the American Foundation for Suicide Prevention (Grant Number SRG-1-043-17).

## Disclaimer

The opinions and assertions expressed herein are those of the author(s) and do not necessarily reflect the official policy or position of the Uniformed Services University, the Department of Defense, or the American Foundation for Suicide Prevention.

## Conflict of Interest

The authors declare that the research was conducted in the absence of any commercial or financial relationships that could be construed as a potential conflict of interest.

## Footnotes

1 https://militarysurvivorstudy.org

## Abbreviation

PTG, Posttraumatic Growth.
